# The Novel Gene *VpPR4-1* from *Vitis pseudoreticulata* Increases Powdery Mildew Resistance in Transgenic *Vitis vinifera* L.

**DOI:** 10.3389/fpls.2016.00695

**Published:** 2016-05-27

**Authors:** Lingmin Dai, Dan Wang, Xiaoqing Xie, Chaohong Zhang, Xiping Wang, Yan Xu, Yuejin Wang, Jianxia Zhang

**Affiliations:** ^1^College of Horticulture, Northwest A&F UniversityYangling, China; ^2^Key Laboratory of Horticultural Plant Biology and Germplasm Innovation in Northwest China, Ministry of AgricultureYangling, China; ^3^State Key Laboratory of Crop Stress Biology in Arid Areas, Northwest A&F UniversityYangling, China

**Keywords:** grapevine, pathogenesis-related protein-4 (PR-4), transformation, powdery mildew, qRT-PCR

## Abstract

Pathogenesis-related proteins (PRs) can lead to increased resistance of the whole plant to pathogen attack. Here, we isolate and characterize a PR-4 protein (VpPR4-1) from a wild Chinese grape *Vitis pseudoreticulata* which shows greatly elevated transcription following powdery mildew infection. Its expression profiles under a number of abiotic stresses were also investigated. Powdery mildew, salicylic acid, and jasmonic acid methyl ester significantly increased the *VpPR4-1* induction while NaCl and heat treatments just slightly induced *VpPR4-1* expression. Abscisic acid and cold treatment slightly affected the expression level of *VpPR4-1*. The *VpPR4-1* gene was overexpressed in 30 regenerated *V. vinifera* cv. Red Globe via *Agrobacterium tumefaciens*-mediated transformation and verified by the Western blot. The 26 transgenic grapevines exhibited higher expression levels of PR-4 protein content than wild-type vines and six of them were inoculated with powdery mildew which showed that the growth of powdery mildew was repressed. The powdery mildew-resistance of Red Globe transformed with *VpPR4-1* was enhanced inoculated with powdery mildew. Moreover, other powdery mildew resistant genes were associated with feedback regulation since VpPR4-1 is in abundance. This study demonstrates that PR-4 protein in grapes plays a vital role in defense against powdery mildew invasion.

## Introduction

Pathogenesis-related proteins (PRs) are involved in higher-plant responses to environment stress. They can be triggered by a large amount of pathogens including fungi, bacteria, and viruses (Van Loon, 1985; Lamb et al., 1989; Linthorst and Van Loon, 1991). Pathogenesis-related proteins were first detected in tobacco leaves under tobacco mosaic virus attack (Van Loon and Van Kammen, 1970). A large number of PRs have since been isolated from a range of other plant species (Sels et al., 2008). Currently, 17 families of PRs have been classified, based on their amino acid sequence similarities, enzymatic activities and other biological properties. These families have been numbered in sequence of discovery (Sels et al., 2008; Sinha et al., 2014). Usually, PRs possess enzymatic functions – so, for example, PR-2 is a 1,3-β-glucanase (Kauffmann et al., 1987), PR-7 has endoproteinase activity, PR-9 is a peroxidase, and PR-10 is a ribonuclease (Lagrimini et al., 1987; Vera and Conejero, 1988; Park et al., 2004; Xu et al., 2010). Moreover, a number of them, e.g., PR-1 and PR-6, have been identified as having antifungal and proteinase inhibitory properties (Green and Ryan, 1972; Niderman et al., 1995). In addition, chitinase activity has been detected in PR-3, PR-4, PR-8, and PR-11 (Van Loon, 1982; Métraux et al., 1988; Melchers et al., 1994), with PR-4 being referred to as chitin-binding proteins.

The PR-4 proteins were first described as wound-inducible proteins and were termed win-1 and win-2 in potato (Stanford et al., 1989; Friedrich et al., 1991). Since then, several PR-4 proteins have been isolated and characterized. PR-4 is not only induced by wounding but also by ethanol, abscisic acid (ABA), salicylic acid (SA), 2,6-dichloroisonicotinic acid, methyl jasmonate (MeJA), sugar starvation, viruses, and fungi (Broekaert et al., 1990; Boll, 1991; Friedrich et al., 1991; Linthorst and Van Loon, 1991; Potter et al., 1993; Melchers et al., 1994; Chevalier et al., 1995; Gregersen et al., 1997; Lee et al., 2001; Bravo et al., 2003; Hamada et al., 2005; Park et al., 2005; Guevara-Morato et al., 2010). The PR-4 proteins possess a Barwin domain in the C-terminal which comes from a barley seed protein related to the wound-induced proteins (Svensson et al., 1992). The secondary structure analysis of the barley seed protein shows the protein contains a large four-stranded antiparallel β-sheet and a small parallel β-sheet (Ludvigsen and Poulsen, 1992a). The binding site of the Barwin domain to the tetramer *N*-acetyl glucosamine was investigated by three-dimensional structural analysis (Ludvigsen and Poulsen, 1992b). The PR-4 proteins are classified into classes I and II based on whether they have an N-terminal Hevein domain or not (Neuhaus et al., 1996). Wheatwin1 and wheatwin2 belong to class II of the PR-4 proteins and show antifungal activity by inhibiting fungal hyphal growth (Caruso et al., 1996, 2001; Huet et al., 2013). Furthermore, wheatwin1 is able to digest RNA from wheat and *Fusarium culmorum* (Caporale et al., 2004). The ribonuclease activity of wheat PR-4 proteins contributes to their antifungal activity (Bertini et al., 2009). In addition, a PR-4 in *Capsicum chinense* has been shown to exhibit deoxyribonuclease and ribonuclease in *in vitro* assays (Guevara-Morato et al., 2010). AtHEL, a class I PR-4 protein of *Arabidopsis*, was found to interact with a fungal lectin having deoxyribonuclease and ribonuclease activities (Bertini et al., 2012; Huet et al., 2013).

Grape production is principally of *Vitis vinifera* cultivars and is also principally geared toward winemaking. Fungal disease is a critical factor in this major international industry, leading to heavy financial penalties due to reductions in both fruit yield and in fruit quality. When infected by fungi, a number of grape PRs are induced, including PR-4 (Kortekamp, 2006). *V. pseudoreticulata* accession Baihe 35-1 is a distinct accession of Chinese wild grape, which possesses high resistance to Erysiphe necator and powdery mildew-induced genes had been isolated from the cDNA library of the high powdery mildew resistant Baihe-35-1 inoculated by Erysiphe necator (Yu et al., 2013). Moreover, *VpPR4* is different from the powdery mildew resistance gene of *V. pseudoreticulata* that we have already studied before (Yu et al., 2013; Dai et al., 2015) or other PR4 gene from similar species in sequence. Powdery mildew can induce abundant expression of PR-4 in *V. vinifera* (Fung et al., 2008). However, the expression of *VpPR4* gene was significant higher than the *PR4* detected in the European grapevine control by cDNA library analyzing in our previous study (Zhu et al., 2012) which suggested that the characterization and expression of *VpPR4* is different compared with *VvPR4*. Moreover, the detailed function of PR-4 proteins in grape is unclear.

This study describes the detection and characterization of a PR-4 protein induced by powdery mildew (Erysiphe necator [Schw.] Burr) in the leaves of *V. pseudoreticulata* Baihe-35-1, and this gene expression profile following exposure to a range of plant hormones and abiotic stresses. The *PR-4* gene was overexpressed in the powdery mildew susceptible variety Red Globe which shows enhanced resistance to powdery mildew and disarranged expression patterns of related defense genes.

## Materials and Methods

### Plant Materials and Stress Treatments

Grapevines (wild Chinese *V. pseudoreticulata* accession Baihe 35-1) were propagated by tissue culture and plantlets were transplanted to pots grown in a culture room (Yu et al., 2013). The inoculation of vine leaves with powdery mildew was carried out as previously described (Guan et al., 2011). The young leaves were inoculated by touching the adaxial surface of the leaves with PM cv. NAFU1 (KJ539202) colonies maintained on the greenhouse-grown grapes. Young grapevine plantlets (*V. pseudoreticulata* Baihe 35-1) 20–25 cm in height were selected for hormone treatments and the leaves were allowed to expand in a greenhouse. Treatments with 100 μM SA, 50 μM MeJA, or 100 μM ABA were imposed by spraying these onto the fully expanded leaves. The solution of SA was 100 μM in distilled water (Wang and Li, 2006), solution of MeJA was 50 μM in 0.1% ethanol (Repka et al., 2004), and the solution of ABA was 100 μM in distilled water (Seong et al., 2007). For the environmental treatments, grapevine plantlets (*V. pseudoreticulata* Baihe 35-1) were incubated in 4°C (cold) or 40°C (hot). For NaCl treatment, the grapevines in pots were watered with 500 mM NaCl (Yang et al., 2012).

### Isolation of Full-length Coding Sequences of *VpPR4-1*

The well-known PR-4s were used to blast search the grape genome on http://www.genoscope.cns.fr/externe/GenomeBrowser/Vitis/. Three homologous sequences were isolated from the grape genome. One was a pseudogene and the other two were used as templates for homology-based cloning of *PR-4s* from *V. pseudoreticulata*. The amplified primers are listed in **Table 1**.

**Table 1:** Primers used for RT-PCR or PCR reactions.

Gene	Primer sequence	Tm (°C)	Amplicon length (bp)
*VpPR4-1* (JN977472) (Construct overexpression plasmid)	Forward: CCCTCGAGATGGAGAGGAGAGGCA	62.5	450
	Reverse: GCTCTAGATTAGTCACCACAGTTC	55.7	
*NPTII* (AF485783) (Detect transgenic lines)	Forward: CCCTGAATGAACTGCAGGACG	56.3	520
	Reverse: CAATATCACGGGTAGCCAACG	54.4	
*VpPR4-1* (RT-PCR)	Forward: TCAGGCAACAGTGAGAATAGTG	53	114
	Reverse: TTAAGATGACCTTGGGCATAGC	53	
*NPR1* (XM_002281439) (RT-PCR)	Forward: GCGGAAAGAGCCCAAGATTA	51.8	107
	Reverse: CTGGTGAGCCTCTTTGCTATT	52.4	
*PR1* (XM_002274239) (RT-PCR)	Forward: GGGTTGTGTAGGAGTCCATTAG	54.8	103
	Reverse: TGTGAGCATTGAGGTAGTCTTG	53	
*PAL* (JN858957) (RT-PCR)	Forward: CTGCGAGAAGGACTTGCTAAA	52.4	95
	Reverse: ACCTTCTGCATCAGTGGATATG	53	
*GAPDH* (EF192466) (RT-PCR)	Forward: TCAAGGTCAAGGACTCTAACACC	55.3	225
	Reverse: CCAACAACGAACATAGGAGCA	52.4	


### Alignment and Phylogenetic Analysis of PR-4 Proteins

Protein sequences of VpPR4-1 and other well-known PR-4s were aligned by DNAman using default parameters. The phylogenetic tree was constructed by Neighbor-joining method using Mega 5.0 software (Tamura et al., 2011). Bootstrap analysis was carried out using 1000 replicates.

### Quantification of Gene Expression by Real-time PCR (qRT-PCR)

A modified SDS/phenol method was used to extract total RNA (Reid et al., 2006). First-strand cDNA synthesis and quantitative RT-PCR were carried out as Hou et al. (2013). The grape *GAPDH* gene (GenBank accession No. EF192466) was amplified as a normalized control. According to Dai et al. (2015), gene expression analysis was carried out by qRT-PCR with a Bio-Rad IQ5 Real-Time PCR Detection System (Hercules, CA, USA) using TaKaRa SYBR Premix Ex TaqTM II (Perfect Real Time). The qRT-PCR reaction was conducted in triplicate following parameters: 95°C for 10 s; 40 cycles of 94°C for 5 s and 60°C for 30 s. The normalized fold expression of RNA was calculated by comparison with the normalized control.

### Binary Vector Construction and Genetic Transformation of Grape

Binary vector construction was carried out as Yu et al. (2013). The *VpPR4-1* gene was PCR cloned in-frame into plasmid pART-CAM-S (Xu et al., 2014) using *Xho* I and *Xba* I restriction enzymes to generate *35S::VpPR4-1*, which contained a kanamycin resistance selective marker. The binary construct was introduced into *Agrobacterium* strain GV3101 using electroporation.

Proembryonic masses of *V. vinifera* L. Red Globe, initiated from immature stamens and maintained from a previous study, were used for genetic transformation with *VpPR4-1*. Transformation was carried out via the *Agrobacterium*-mediated transformation system as described previously (Zhou et al., 2014; Dai et al., 2015).

### Western Blotting

Total protein was extracted using phenol-based protocols as described previously (Vincent et al., 2006). The protein was fractionated by 5–10% SDS-PAGE and blotted to polyvinyl difluoride membranes (Roche) using a semi-dry blot apparatus as described by the manufacturer (Bio-Rad). The purified polyclonal antisera antibody of VpPR4-1 protein obtained from New Zealand rabbits immunized by purification of the prokaryotic expressed VpPR4-1 protein in our previously study (data not shown), and the polyclonal antisera was used at 1:2,000 dilution and secondary goat anti-rabbit IgG (TransGen Biotech) conjugated with alkaline phosphatase at 1:5,000 dilution. Detection was carried out using Pierce ECL Western Blotting Substrate (Thermo scientific) and imaged with Image Lab Software (Bio-Rad).

### Trypan Blue Staining

The trypan blue staining is used for observing powdery mildew hyphal development and carried out according to Xiao et al. (2003) with minor modification. Briefly, grape leaves were picked and stained with the Trypan blue staining solution in a boiling water bath for 15 min and then allowed to rest at room temperature for 24 h. The samples were then incubated in chloral hydrate solution for 2–6 h at room temperature with the chloral hydrate saturated solution changed once and then incubated for a further 8 h at room temperature. The chloral hydrate solution was then removed and the tissue immersed in 70% glycerol and examined using a compound light microscope.

### Statistical Analysis

Experimental results in **Figures 2**, **5**, and **7** are mean values of triplicate experiments; error bars in **Figures 2** and **5** indicate standard deviations (SD). Mean values and SD were calculated using SPASS 18.0 software (SPSS Inc, Chicago, IL, USA).

## Results

### Sequence Analysis of *VpPR4-1* from Wild Chinese *V. pseudoreticulata*

**FIGURE 1:**
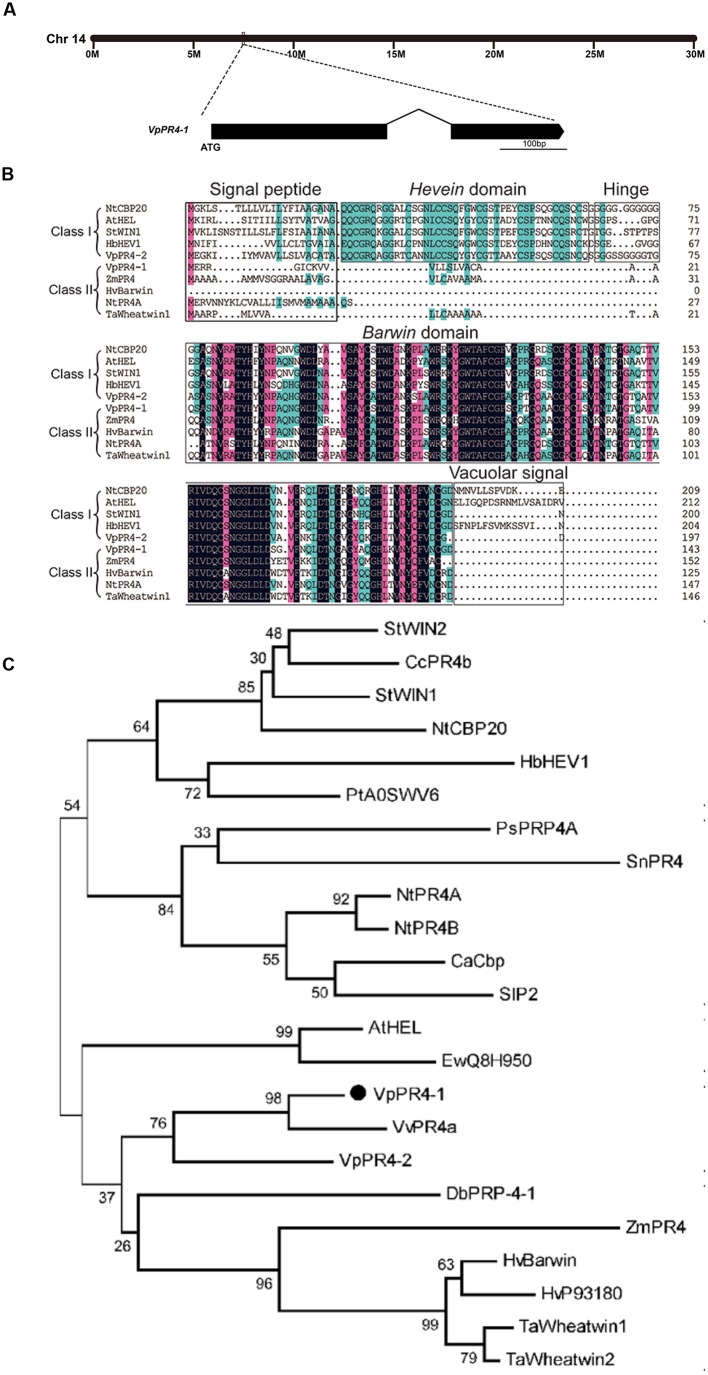
**Phylogenetic analysis and alignment of PR-4 proteins. (A)** Location on the chromosome and schematic representation of the *VpPR4-1* gene. **(B)** Sequence alignment of VpPR4-1. The conserved domains are shown above the sequences. Identical amino acids are shaded with black, and similar amino acids are shaded with pink (<100 and ≥75%) or blue (<75 and ≥50%). **(C)** Phylogenetic analysis of various PR-4-1 proteins. Class I, subgroup of PR-4 proteins which contain a *Hevein* domain. Class II, subgroup of PR-4s which do not have a *Hevein* domain. Accession numbers are: VpPR4-1 (AEW12795; Vp, *Vitis pseudoreticulata*), VpPR4-2 (KP274873), StWIN2 (NP_001275628; St, *Solanum tuberosum*), CcPR4b (BAD11073; Cc, *Capsicum chinense*), StWIN1 (XP_006347743), NtCBP20 (AAB29960; Nt, *Nicotiana tabacum*), HbHEV1 (P02877; Hb, *Hevea brasiliensis*), PtA0SWV6 (ABK63195; Pt, *Populus tremula* × *Populus alba*), PsPRP4A (AAF61434; Ps, *Pisum sativum*), SnPR4 (CAA87070; Sn, *Sambucus nigra*), NtPR4A (XP_009763689), NtPR4B (XP_009614804), CaCbp (AFN21550; *Capsicum annuum*), SlP2 (NP_001234083; Sl, *Solanum lycopersicum*), AtHEL (NP_187123; At, *Arabidopsis thaliana*), EwQ8H950 (BAC16357; Ew, *Eutrema wasabi*), VvPR4a (AAC33732; Vv, *Vitis vinifera*), DbPRP-4-1 (AAB94514; Db, *Dioscorea bulbifera*), ZmPR4 (AFW60484; Zm, *Zea mays*), HvBarwin (BAK04328; Hv, *Hordeum vulgare*), HvP93180 (CAA71774), TaWheatwin1 (O64392; Ta, *Triticum aestivum*), and TaWheatwin2 (O64393).

The full-length cDNA of *VpPR4-1* was homologous cloned from the cDNA library of high powdery mildew resistant *V. pseudoreticulata* Baihe 35-1 inoculated by powdery mildew (Zhu et al., 2012). The *VpPR4-1* gene is on the chromosome 14 of the whole genome of PN40024 (Jaillon et al., 2007), it is 432 base pairs (bp) long and codes for a unique polypeptide of 143 amino acids with two exons (**Figure 1A**) and possesses a Barwin domain (**Figure 1B**).

Numerous PR-4 proteins have been detected from a variety of different species. To understand the evolutionary relationships between these PR-4 proteins, alignment and phylogenetic analyses were carried out. Alignment analysis of PR-4s showed that all contained a Barwin domain. However, PR-4s can be divided into two classes on the basis of whether they have a Hevein domain. VpPR4-1 fell into class II according to this classification scheme (**Figure 1B**). Phylogenetic analysis indicated that the PR-4s fell into two subgroups. The VpPR4-1 is closely referred to the PR-4 protein (BAK04328) from *Hordeum vulgare* subsp. at the amino acid level (**Figure 1C**). Vulgare sharing 79.3% of sequence identity (Matsumoto et al., 2011). For other PR-4 proteins used for alignment and phylogenetic analyses, the sequence identities ranged from 61.7 to 76.7%.

### Induction of *VpPR4-1* by Powdery Mildew, Exogenous Hormones, and Abiotic Stresses

**FIGURE 2:**
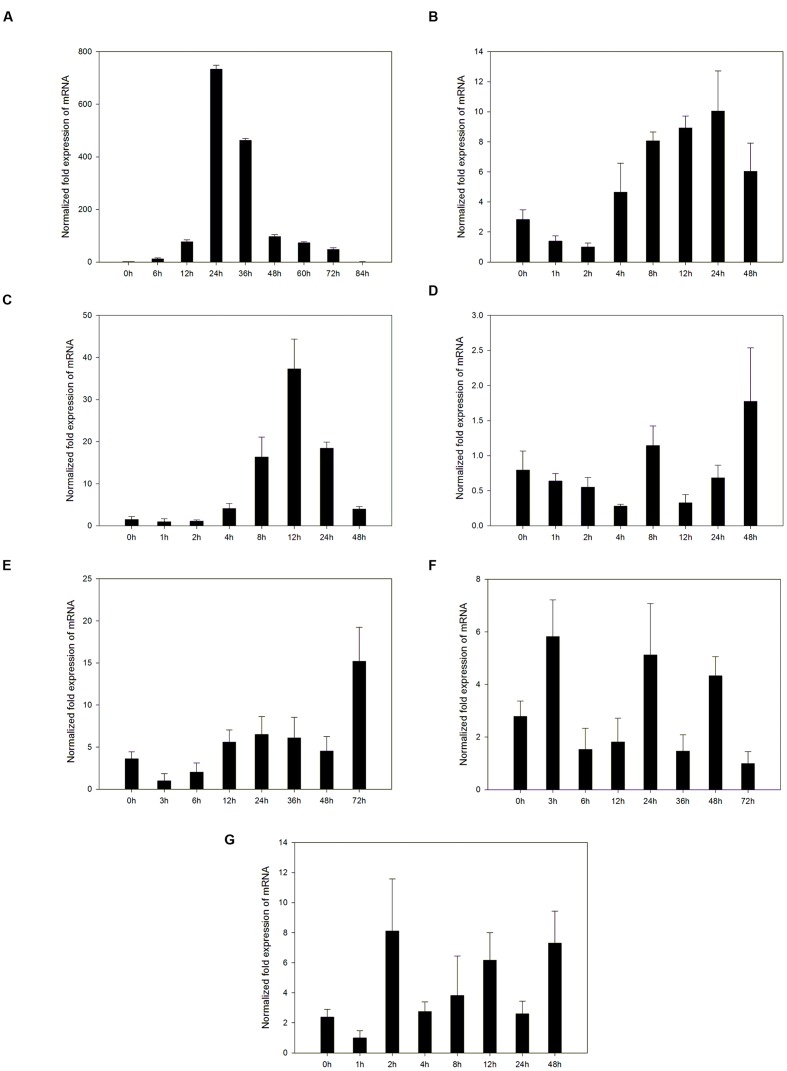
**Accumulation of mRNA of *VpPR4-1* was assessed by quantitative RT-PCR in leaves under a number of stresses. (A)** Expression profiles of *VpPR4-1* infection with powdery mildew. **(B)** Expression profiles of *VpPR4-1* under SA treatment. **(C)** Expression profiles of *VpPR4-1* under MeJA treatment. **(D)** Expression profiles of *VpPR4-1* under ABA treatment. **(E)** Expression profiles of *VpPR4-1* under NaCl treatment. **(F)** Expression profiles of *VpPR4-1* under 4°C treatment. **(G)** Expression profiles of *VpPR4-1* under 42°C treatment. Error bars indicate SD, from three independent experiments.

PRs are usually induced by pathogen attack. Expression of the *VpPR4-1* induced by powdery mildew was demonstrated in the powdery mildew resistant genotype *V. pseudoreticulata* Baihe 35-1 (**Figure 2A**). Under normal conditions, the transcript level of *VpPR4-1* was very low. Upon infection with powdery mildew *VpPR4-1* transcript levels increased significantly, reaching a maximum 24 h post inoculation (hpi) and then declined. The induction increase of *VpPR4-1* transcript level was very high, showing a nearly 800-fold increase compared with the normal level.

To determine the upstream pathway of *VpPR4-1*, exogenous hormones and abiotic stresses were imposed for RT-PCR. The *VpPR4-1* responded differently to the applications of three exogenous hormones and three abiotic stresses (**Figures 2B–G**). The PR gene responds differently to different stresses in *V. pseudoreticulata* Baihe 35-1. ABA and cold treatment slightly affected the expression level of *VpPR4-1*. SA increased the level *VpPR4-1* induction, which peaked at 24 hpi. MeJA increased *VpPR4-1* induction strongly, with a peak at 12 hpi. This was about a 33-fold increase above normal. However, following NaCl and heat treatments, *VpPR4-1* expression increased only about 3-fold.

### Molecular Analysis of Overexpression

**FIGURE 3:**
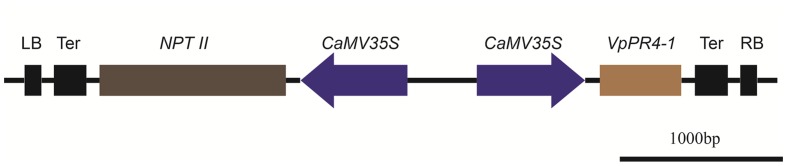
**Physical maps of plant transformation vector *VpPR4-1*.** LB, Left border of T-DNA; Ter, terminator; *NPTII*, neomycin phosphotransferase gene; *CaMV35S*, Cauliflower mosaic virus 35S promoter; *VpPR4-1*, pathogen-related protein gene from *Vitis pseudoreticulata* Baihe 35-1; RB, Right border of T-DNA.

**FIGURE 4:**
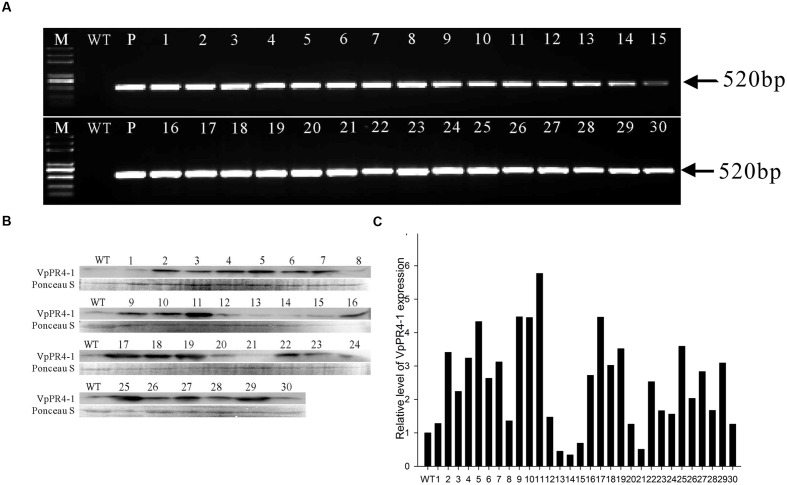
**Molecular analysis of transgenic *Vitis vinifera* L. Red Globe by overexpression *VpPR4-1* from *V. pseudoreticulata*. (A)** PCR analysis of *NPTII* from transgenic *V. vinifera* L. Red Globe of *VpPR4-1*. **(B)** Western blotting analysis of transgenic *V. vinifera* L. Red Globe by overexpression *VpPR4-1.* VpPR4-1, showing the Western blotting of VpPR4-1 in transgenic lines; Ponceau S, showing the protein stained by Ponceau S. **(C)** Quantitative analysis for VpPR4-1 protein expression of Western blotting analysis of transgenic *V. vinifera* L. Red Globe by overexpression *VpPR4-1* **(B)** were performed by Image lab 4.1 software. WT, wild-type line; 1–30, 30 transgenic lines of *VpPR4-1*.

To further understand the functional response of *VpPR4-1* in grapevine, in relation to powdery mildew attack, functional analyses of *VpPR4-1* in transgenic *V. vinifera* L. Red Globe were carried out with the overexpression construct *VpPR4-1* (**Figure 3**) via *Agrobacterium*-media transformation (Supplementary Figure 1). A total of 30 independent transgenic Red Globe plantlets were obtained. Genomic DNA was extracted from the transgenic grapevines of *VpPR4-1* and used to amplify the 520 bp fragment of *NPTII*. *NPTII* was detected in all transgenic lines and positive plasmid control, whereas no amplification was detected in the wild-type (WT) plant line (**Figure 4A**). The expression profiles of VpPR4-1 in transgenic plant lines were studied using the Western-blot method. Total proteins were extracted from the WT plant line and transgenic plant lines of *VpPR4-1* under the same growth condition and blotting using polyclonal antibody. Twenty-six of *VpPR4-1* transgenic lines showed higher expression levels of VpPR4-1 than the WT plant line (**Figures 4B,C**). However, expression levels of VpPR4-1 of transgenic lines 13, 14, 15, and 21 were lower than the WT grape. Transgenic lines *VpPR4-1*-4 and *VpPR4-1*-5 showed relatively higher levels of PR4 protein and were selected for further experiments.

### Enhanced Tolerance to Powdery Mildew of Transgenic Plants

**FIGURE 5:**
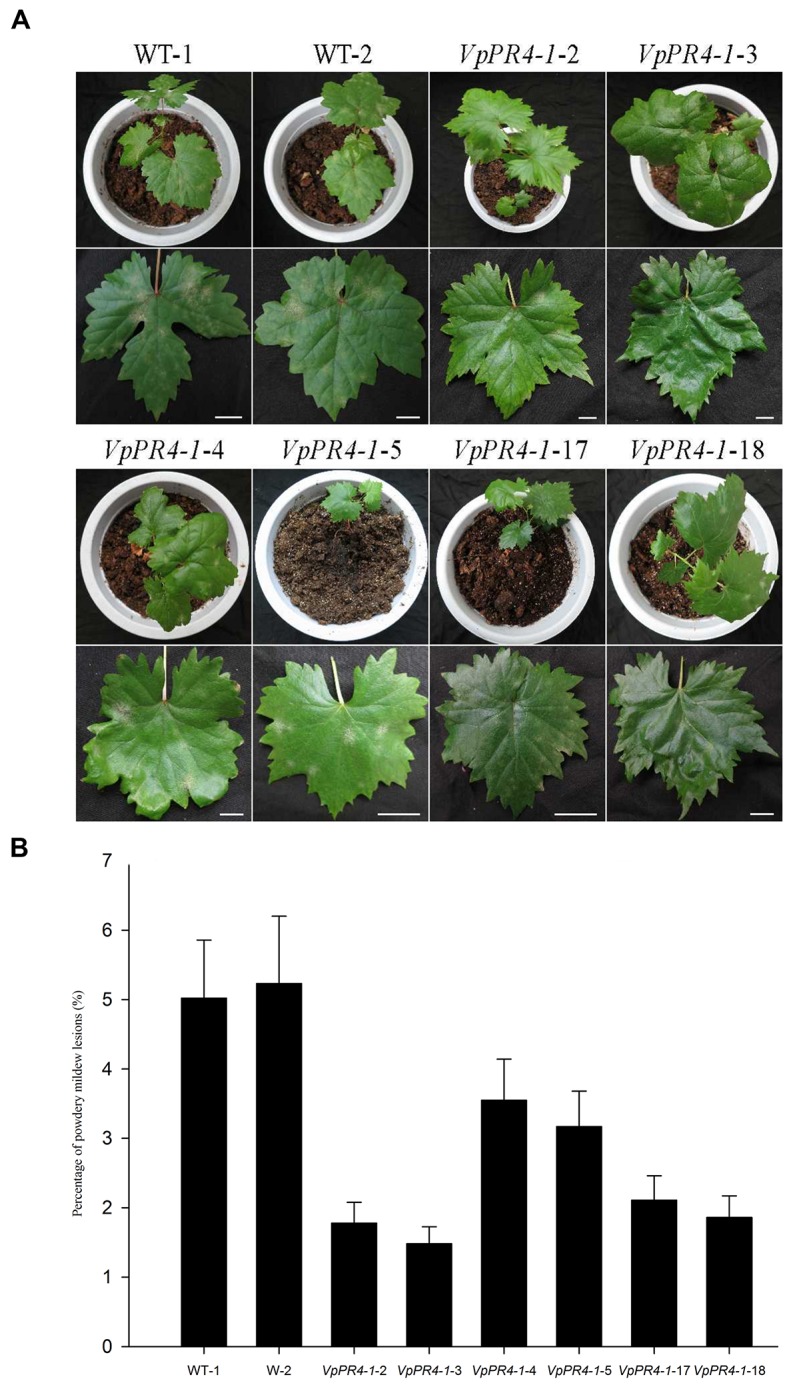
**Powdery mildew-tolerance analysis for transgenic *Vitis vinifera* L. Red Globe by overexpression *VpPR4-1* from *V. pseudoreticulata*. (A)** Images were taken 12 days after inoculation. **(B)** Percentages of powdery mildew lesions were analyzed by ImageJ 1.43q software. Error bars indicate SD of three replications. WT-1 and WT-2, wild-type lines; *VpPR4*-1-2, -3, -4, -5, -17, and -18, transgenic lines of *VpPR4-1*. Scale bar = 2 cm.

**FIGURE 6:**
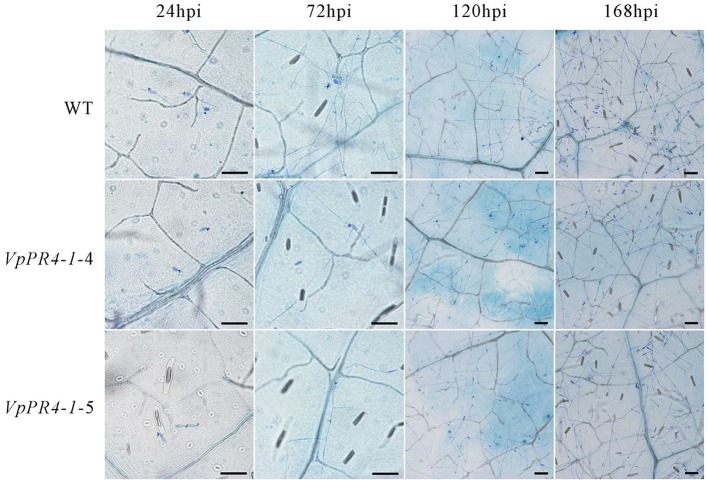
**Powdery mildew invaded the leaves of transgenic *Vitis vinifera* L. Red Globe by overexpression *VpPR4-1* from *V. pseudoreticulata*.** WT, wild-type line; *VpPR4-1*-4 and *VpPR4-1*-5, transgenic line 4 and 5 of *VpPR4-1*, respectively. Scale bar = 100 μm.

To confirm that *VpPR4*-1 actually participates in antifungal activity, we compared the growth rates of powdery mildew hyphae between the six overexpression lines of the higher PR4 protein level and WT plants after powdery mildew infection. Hyphal growth during a 12-day period infection with powdery mildew revealed enhanced tolerance of transgenic plants to the fungus than of none transgenic plants. The *VpPR4-1*-2, *VpPR4-1*-3 showed better resistant than other transgenic lines (**Figure 5**). To investigate the growth of powdery mildew, light microscopy with trypan blue staining was used as previously described (Xiao et al., 2003). Images at 24, 72, 120, and 168 hpi are shown in **Figure 6**. In the WT lines, more hyphal growth was observed than in the transgenic plants, and the overexpression lines significant reduced powdery mildew hyphal growth not inhibited the fungal completely (**Figure 6**).

**FIGURE 7:**
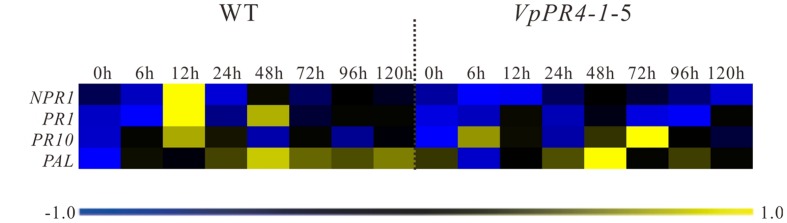
**qPCR expression analysis for disease-resistance associated genes in leaves of transgenic *Vitis vinifera* L. Red Globe by overexpression *VpPR4-1* from *V. pseudoreticulata* under powdery mildew treatment.** WT, wild-type grapevines; *VpPR4-1*-5, transgenic line 5 containing *VpPR4-1*. The color heat map was created according to the standardized RT-PCR expression data by median normalization method using MeV 4.9.0 software, and the color coded showed the differences in gene expression (see key, bottom). The color scale represents relative expression levels: blue represents decreased transcript abundance and yellow represents increased transcript abundance.

To investigate expression changes of disease-resistance associated genes in the transgenic lines, *NPR1*, *PR1*, *PR10*, and *PAL* genes transcript levels were measured in the *VpPR4-1-4*, *VpPR4-1-5*, and WT lines inoculated with powdery mildew. These showed that the expression of disease-resistance associated genes was induced by powdery mildew and peaked between 12 and 48 hpi, before returning to the low baseline level in the WT line by 72 hpi. However, compared with WT grapes, the defense related genes were disturbed to different degrees by the *VpPR4-1* overexpression when inoculated with powdery mildew. In transgenic lines, the expression of the *NPR1*, *PR1*, and *PAL* genes displayed a lower transcription level, and the peak of the *PR10* gene transcript was delayed when inoculated with powdery mildew (**Figure 7**). Specifically, in the WT plants, *NPR1* was induced and reached a 30-fold peak at 12 hpi but there was no significant change in the overexpression lines. Correspondingly, in WT, *PR1* showed a double peak expression pattern while in the *VpPR4-1* transgenic lines this was suppressed and showed a delayed response. However, *PAL* was not materially affected since both the WT and overexpression lines showed a tendency to rise at first but then decline later.

Further gene function and the role of powdery mildew resistance of *VpPR4-1* including the disease resistance tests in the vineyard will be studied in our future study based on the current work and the transgenic lines of *VpPR4-1* obtained.

## Discussion

Powdery mildew is one of the most damaging fungal diseases of the European grapevines. Since powdery mildew resistance is regulated by multi-gene, grapevine is still recalcitrant to powdery mildew resistance and the genes from the cDNA library of the high powdery mildew resistant Baihe 35-1 is worth researching. In our study, detection and characterization of a PR-4 protein induced by powdery mildew in the leaves of *V. pseudoreticulata* Baihe 35-1 were detected, sequence analyses and expression patterns of *VpPR4-1* were carried out. Finally, *VpPR4-1* was overexpressed in the powdery mildew susceptible variety Red Globe to exhibit its resistance to powdery mildew.

### VpPR4-1 Belongs to the Class II PR-4 Proteins

*Vitis pseudoreticulata* shows high resistance to powdery mildew. The PR-4 proteins, VpPR4-1, were isolated from *V. pseudoreticulata* and characterized. VpPR4-1 has previously been cloned from expressed sequence tags (Shi et al., 2010). The PR-4 protein precursor has a short signal peptide and the mature protein format undergoes a strict cutoff. Based on the lack of a *Hevein* domain near the N terminal, VpPR4-1 belongs to class II. The class II PR-4 proteins are proposed to have evolved from the class I PR-4 proteins because of the highly conserved sequences in the C terminal and the deletion of the only *Hevein* domain (Friedrich et al., 1991; Ponstein et al., 1994). And sequence alignment and phylogenetic analysis indicate that VpPR4-1 belongs to class II and shows a high level of sequence conservation with these well characterized class II PR-4 proteins.

### *VpPR4-1* Was More Strongly Induced by Powdery Mildew than by the Other Stresses

*PR-4* mRNA expression level in grape increased during powdery mildew incubation. However, the induction patterns in susceptible and non-susceptible cultivars are quite different (Fung et al., 2008). Wild Chinese grape *V. pseudoreticulata* is an accession having high resistance to powdery mildew. The *PR-4* gene expression levels are significantly higher in leaves of *V. pseudoreticulata* following inoculation with powdery mildew than that under normal conditions. Nevertheless, SA, MeJA, ABA, cold, heat, or NaCl stresses do not stimulate *VpPR4-1* to a similar extent to powdery mildew attack although they do lead to PR-4 up-regulation. A possible explanation for the expression profile is that powdery mildew regulates *VpPR4-1* in an independent or crossover pathway to these six abiotic stresses. It is well known that SA and JA have positive roles in disease resistance, while ABA has a negative role (Kunkel and Brooks, 2002; Mauch-Mani and Mauch, 2005). In this study, *VpPR4-1* was induced by SA and MeJA (by 3- to 30-fold) but was insensitive to ABA. A different result was observed with ABA treatment in rice which shows threefold to ninefold change in the expression levels of the *PR-4* genes. The expression profiles of *VpPR4-1* were also induced by a number of environment stresses (including by salt, cold and heat shock) but the responses were slow. Together, these results suggest that VpPR4-1 has a positive role in stress responses in *V. pseudoreticulata*, which may be regulated by SA- and JA networks. Moreover, our results are consistent with Wang et al. (2011).

### Overexpression of VpPR4-1 Increases Powdery Mildew Resistance of *V. vinifera*

The Ribonuclease activity of PR-4 proteins has been reported a number of times and many experiments have confirmed that they can repress fungal growth *in vitro* (Bai et al., 2013; Vaghefi et al., 2013; Menezes et al., 2014). To expand our knowledge about PR-4 proteins and create resistant table-grape germplasm, we overexpressed the *VpPR4-1* gene in *V. vinifera* cv. Red Globe. In spite of PR-4 proteins having been implicated in stress responses (Wang et al., 2011), no direct evidence has been reported previously showing that the PR-4 proteins participate in powdery mildew resistance in an important food crop such as grapevines. The *VpPR4-1* gene was overexpressed in 30 regenerated *V. vinifera* cv. Red Globe via *Agrobacterium tumefaciens*-mediated transformation and confirmed by PCR of *NPTII*. The 26 transgenic grapevines exhibited higher expression levels of PR-4 protein content than WT vines verified by the Western blot. It is probably that the target gene between the right and left border of T-DNA in *Agrobacterium* was randomly transformed into the chromosome of host cell via *Agrobacterium tumefaciens*-mediated transformation and even multicopy of target genes transformation which could make gene expression different from each other or even gene silencing in transgenic lines (Bouquet et al., 2007), the expression of PR4 protein were not the same in all transgenic lines and the transgenic lines *VpPR4*-1-13, 14, 15, and 21 were lower than the WT grape, that is consistent with other gene transformation of grapevine (Zhou et al., 2014; Dai et al., 2015; Cheng et al., 2016). To confirm that VpPR4-1 actually participates in antifungal activity, we compared the growth rates of powdery mildew hyphae between the six overexpression lines of the higher PR4 protein level and WT plants after powdery mildew infection. The overexpression lines showed relatively high gene expression levels (**Figures 4B,C**) and significant reduced powdery mildew hyphal growth but not inhibited the fungal completely (**Figure 6**) which is probably due to powdery mildew resistance is regulated by multi-gene complex network and not by a dominant gene (Fung et al., 2008), and the result is similar with Bertini et al. (2012). Various related genes exhibit different expressions under normal or stress conditions when genes are overexpressed *in vivo* (Tillett et al., 2012; Xu et al., 2014). The PR-4 proteins are pathogenesis-related proteins involved in pathogen defense response. Since powdery mildew resistance is regulated by multi-gene of SA and/or MeJA pathways and other network (Fung et al., 2008), it is supposed that the significantly higher expression of PR4 protein may lead to affect the expression of other pathogen relative genes which also play important role in powdery mildew resistance network. Therefore, we detected the influences of overexpression of *VpPR4-1* on related genes transcripts, including: *NPR1*, *PR1*, and *PAL*. In the overexpression lines, the expression level of *NPR1* which plays a positive role in inducible plant disease resistance, was obviously down-regulated. The results may be associated with feedback regulation since *PR-4* is in abundance. Accordingly, *PR1* was also suppressed to some extent. It was different for *PAL*, this key enzyme catalyzes the rate-limiting step of phenylpropanoid biosynthesis in plants, that occurred negligibly during the long incubation period. And it is supposed that the higher expression of PR4 protein leading to the suppression expression of other disease resistant genes which resulted in that *VpPR4-1*-4 and *VpPR4-1*-5 with higher PR4 levels were not so resistant for powdery mildew as *VpPR4-1*-2 (**Figure 5**). Though numerous details for the regulation mechanism of *VpPR4-1* in bestowing resistance to abiotic and biotic stress remains unclear (Wang et al., 2011), the twin facts that *VpPR4-1* is responsive to powdery mildew inoculation and that the *VpPR4-1* overexpression lines showed enhanced powdery mildew resistance in *V. vinifera* cv. Red Globe indicate that they *do* nevertheless play an important role in increasing fungal resistance.

## Author Contributions

LD had made the substantial contributions to the conception, design of the work, and the acquisition, analysis. DW had done interpretation of data for the work. XX did the revising manuscript critically for important intellectual content. JZ did drafting the work of the manuscript. YW gave final approval of the version to be published; XW, YX, and CZ made the agreement to be accountable for all aspects of the work in ensuring that questions related to the accuracy or integrity of any part of the work are appropriately investigated and resolved.

## Conflict of Interest Statement

The authors declare that the research was conducted in the absence of any commercial or financial relationships that could be construed as a potential conflict of interest.
