# Supplementary material for: The Novel Gene VpPR4-1 from Vitis pseudoreticulata Increases Powdery Mildew Resistance in Transgenic Vitis vinifera L
Source: Front Plant Sci. 2016 May 27;7:695. doi: 10.3389/fpls.2016.00695 (PMC4882328; doi:10.3389/fpls.2016.00695)
Supplement: Supplementary file 1 [file Data_Sheet_1.DOCX]

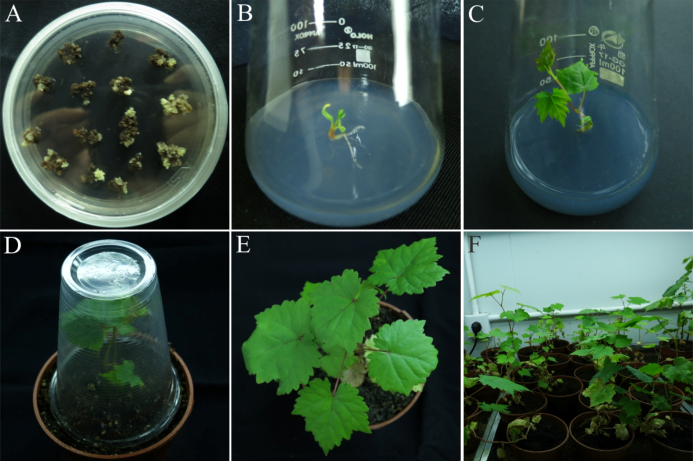


Supplemental figure 1 System for transformation of *VpPR4-1* in *Vitis vinifera* cv. Red Globe via *Agrobacterium*-mediated system. (A) Proembryonic masses selected on medium containing carbenicillin, cefotaxime and 75 mg L^-1^ Kan after co-culture and disinfection. (B) and (C) Kan-resistant SEs and plantlet formed. (D) and (E) Plantlets cultured in pots for hardening in the growth chamber. (F) Grapevines in the greenhouse.
